# Modeling of Oxygen Transport and Cellular Energetics Explains Observations on In Vivo Cardiac Energy Metabolism

**DOI:** 10.1371/journal.pcbi.0020107

**Published:** 2006-09-15

**Authors:** Daniel A Beard

**Affiliations:** 1 Department of Physiology, Medical College of Wisconsin, Milwaukee, Wisconsin, United States of America; 2 Biotechnology and Bioengineering Center, Medical College of Wisconsin, Milwaukee, Wisconsin, United States of America; University of Auckland, New Zealand

## Abstract

Observations on the relationship between cardiac work rate and the levels of energy metabolites adenosine triphosphate (ATP), adenosine diphosphate (ADP), and phosphocreatine (CrP) have not been satisfactorily explained by theoretical models of cardiac energy metabolism. Specifically, the in vivo stability of ATP, ADP, and CrP levels in response to changes in work and respiratory rate has eluded explanation. Here a previously developed model of mitochondrial oxidative phosphorylation, which was developed based on data obtained from isolated cardiac mitochondria, is integrated with a spatially distributed model of oxygen transport in the myocardium to analyze data obtained from several laboratories over the past two decades. The model includes the components of the respiratory chain, the F_0_F_1_-ATPase, adenine nucleotide translocase, and the mitochondrial phosphate transporter at the mitochondrial level; adenylate kinase, creatine kinase, and ATP consumption in the cytoplasm; and oxygen transport between capillaries, interstitial fluid, and cardiomyocytes. The integrated model is able to reproduce experimental observations on ATP, ADP, CrP, and inorganic phosphate levels in canine hearts over a range of workload and during coronary hypoperfusion and predicts that cytoplasmic inorganic phosphate level is a key regulator of the rate of mitochondrial respiration at workloads for which the rate of cardiac oxygen consumption is less than or equal to approximately 12 μmol per minute per gram of tissue. At work rates corresponding to oxygen consumption higher than 12 μmol min^−1^ g^−1^, model predictions deviate from the experimental data, indicating that at high work rates, additional regulatory mechanisms that are not currently incorporated into the model may be important. Nevertheless, the integrated model explains metabolite levels observed at low to moderate workloads and the changes in metabolite levels and tissue oxygenation observed during graded hypoperfusion. These findings suggest that the observed stability of energy metabolites emerges as a property of a properly constructed model of cardiac substrate transport and mitochondrial metabolism. In addition, the validated model provides quantitative predictions of changes in phosphate metabolites during cardiac ischemia.

## Synopsis

To function properly over a range of work rates, the heart must maintain its metabolic energy level within a range that is narrow relative to changes in the rate of energy utilization. Decades of observations have revealed that in cardiac muscle cells, the supply of adenosine triphosphate (ATP)—the primary currency of intracellular energy transfer—is controlled to maintain intracellular concentrations of ATP and related compounds within narrow ranges. Yet the development of a mechanistic understanding of this tight control has lagged behind experimental observation. This paper introduces a computational model that links ATP synthesis in a subcellular body called the mitochondrion with ATP utilization in the cytoplasm, and reveals that the primary control mechanism operating in the system is feedback of substrate concentrations for ATP synthesis. In other words, changes in the concentrations of the products generated by the utilization of ATP in the cell (adenosine diphosphate and inorganic phosphate) effect changes in the rate at which mitochondria utilize those products to resynthesize ATP.

## Introduction

Over 30 years ago, Neely et al. [1] showed that adenosine triphosphate (ATP), adenosine diphosphate (ADP), and adenosine monophosphate (AMP) concentrations are maintained at essentially constant concentrations with changes in cardiac work in the isolated perfused heart. Twenty years ago, Balaban et al. [2] observed that energetic phosphate concentrations measured in vivo using NMR spectroscopy remain effectively constant over a range of cardiac workloads. These observations contradicted earlier models of the control of mitochondrial metabolism, which assumed that the rate of oxidative phosphorylation was regulated primarily by the availability of ADP [3,4]. Specifically, the cytoplasmic ADP concentration and the ratio of creatine phosphate (CrP) to ATP concentration was found to be approximately constant over a range of workload and rates of oxygen consumption in canine hearts [2,5]. To date, a credible validated biophysical model of the in vivo regulation of oxidative phosphorylation that explains the observed phenomena has not been established. Such a model would provide a theoretical basis for understanding how mitochondrial metabolism is regulated in response to changing ATP turnover rate while maintaining homeostatic concentrations of ATP, ADP, and CrP. Such a model would also form the basis of quantitative studies of the regulation of phosphate metabolites oxidative phosphorylation in the failing heart and other pathophysiological situations [6].

In this work, a detailed model of cardiac oxidative phosphorylation that was developed based on an extensive set of data obtained from isolated mitochondria [7] is integrated with an axially distributed model of oxygen transport and exchange with tissue [8]. The mitochondrial model includes the components of the respiratory chain, the F_0_F_1_-ATPase, adenine nucleotide translocase, and the mitochondrial phosphate transporter. The mitochondrial model is integrated with a model of ATP, ADP, AMP, and CrP metabolism in the cytoplasm, including the reactions of adenylate kinase, creatine kinase, and ATP consumption. Oxygen transport is governed by advection of blood in capillaries, passive permeation between blood and interstitial fluid and between interstitial fluid and cardiomyocytes, and metabolic consumption at Complex IV of the respiratory chain. An empiric relationship between cardiac perfusion and ATP consumption rate is determined by comparing model simulations to data published by Katz et al. [5]. The behavior of the resulting integrated model is compared to datasets published by Katz et al. [5], Portman et al. [9], and Zhang et al. [10], which report on in vivo changes in cardiac phosphate metabolites in response to changes in workload and to graded ischemia.

## Results

### Overview of Oxygen Transport

Oxygen transport and energetic metabolism in cardiac tissue are simulated using a three-region one dimensionally distributed model [8,11], illustrated in Figure 1. The model assumes that within the capillary, interstitial space, and cellular (myocyte) space, the concentrations of oxygen and other metabolites vary primarily along the length of the capillary. Advective transport is modeled in the capillary region; the interstitial and cellular spaces are assumed to be stagnant (nonflowing). The cellular region is further subdivided into cytoplasmic and mitochondrial compartments, as described below. The full set of model variables is listed in Table 1, along with a brief description of the variables and the units associated with each variable. Note that oxygen concentrations are expressed as mass per unit volume, while the other intracellular species are expressed as mass per unit water volume in a given region. Each variable in the model is a function of distance along the capillary *x* and time *t*.

**Figure 1:**
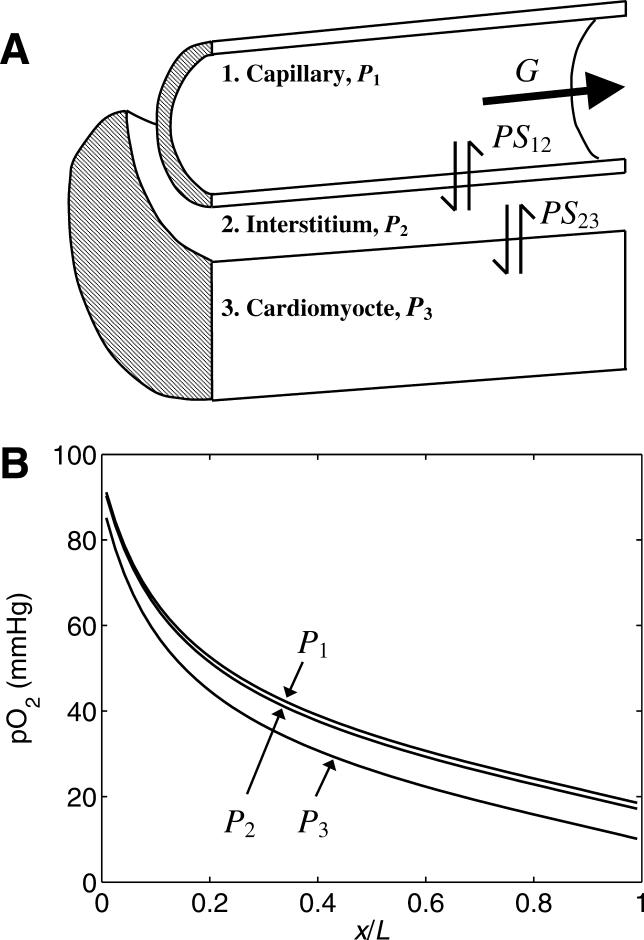
Axially Distributed Blood–Tissue Exchange Model for Oxygen Transport (A) A three-region one dimensionally distributed model for oxygen transport is diagrammed. The three regions correspond to capillary, interstitium, and cell (myocyte). Blood flows in the capillary region. Oxygen is transported via passive permeation between the capillary, interstitial, and myocyte regions. (B) Predicted capillary, interstitial, and cellular Po_2_ are plotted as functions of *x*/*L,* the normalized distance along the capillary. Simulation parameters are *TPP* = 15 mM, *G* = 0.75 ml min^−1^ (g of tissue)^−1^, and *J*_AtC_ = 0.45 mmol s^−1^ (l cell)^−1^. The input arterial Po_2_ is assumed to be 100 mm Hg. The predicted rate of oxygen consumption and oxygen extraction are found to be 5.3 μmol min^−1^ (g of tissue)^−1^ and 76%, respectively.

**Table 1:**
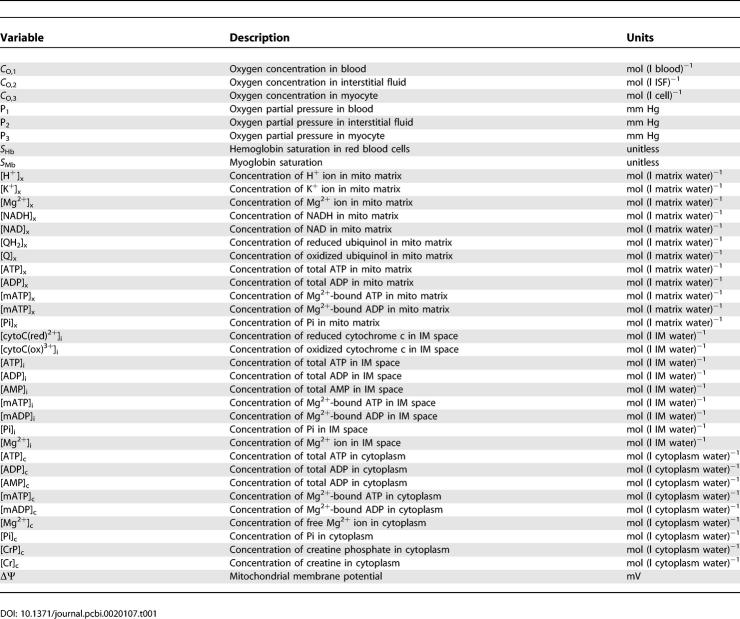
Model Variables

A representative model-predicted steady-state oxygen concentration profile is illustrated in Figure 1B, which plots the capillary, interstitial, and cellular Po_2_ as functions of *x*/*L*, the normalized distance along the capillary. Partial pressures decrease from the arterial (*x* = 0) to the venous (*x* = *L*) end of the capillary and decrease from the capillary region to the cellular region.

To obtain the results illustrated in Figure 1B, the total pool of exchangeable phosphate in the cell (see Equation 26 in Materials and Methods) was set to 15 mM, flow was set to *G* = 0.75 ml min^−1^ (g of tissue)^−1^, and the rate of ATP consumption was set to *J*_AtC_ = 0.45 mmol s^−1^ (l cell)^−1^. For all simulations, the input arterial Po_2_ was assumed to be 100 mm Hg. For these values, the rates of oxygen consumption and oxygen extraction were found to be 5.3 μmol min^−1^ (g of tissue)^−1^ and 76%, respectively, values which are consistent with a moderate rate of cardiac work [12,13].

### Relationship between Flow and ATP Consumption

To simulate the behavior of the model at different workloads, it is necessary to define a relationship between workload and coronary perfusion. In Katz et al. [5], cardiac flow and the rate of cardiac oxygen consumption, MVO_2_, are measured under different conditions. Yet in the integrated model, MVO_2_ is not set as an input parameter; MVO_2_ is computed as a function of the rate of ATP consumption *J*_AtC_. To determine a relationship between *G* and *J*_AtC_, the model was fit to data from Tables 2, 3, and 4 of Katz et al. [5], which report *G* and MVO_2_ for paced hearts, hearts stimulated with epinephrine, and hearts stimulated with phenylephrine. For entry in these tables, the value of *J*_AtC_ was varied until the model-predicted MVO_2_ was equal to the given experimental measure. The resulting data are plotted in Figure 2A. The observed *G* and *J*_AtC_ values approximately obey the linear relationship *G* = 0.3326 + (2.315)*J*_AtC_, where *J*_AtC_ is expressed in units of mmol s^−1^ (l cell)^−1^ and *G* is computed in units of ml min^−1^ (g of tissue)^−1^. The linear fit between the flow and ATP consumption rate is indicated by the straight line in the figure. This relationship is used to specify a value of *G* to use for simulating the system behavior at a given rate of *J*_AtC_ in the following section.

**Figure 2:**
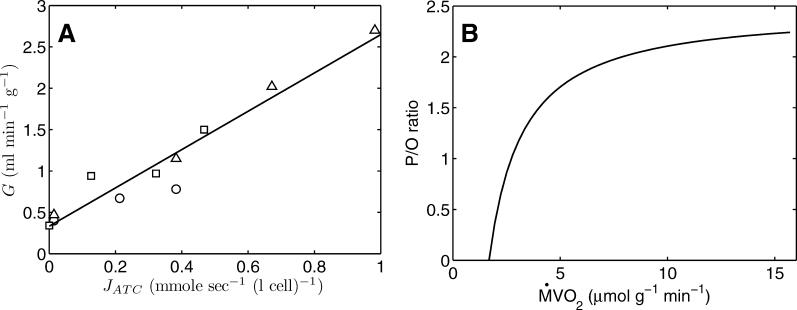
Relationships between ATP Consumption Rate, Blood Flow, and Rate of Oxygen Consumption in Cardiac Tissue (A) Plot of coronary flow from Tables 2, 3, and 4 of Katz et al. [5] as a function of predicted rate of ATP consumption, *J*_AtC_. The values of *J*_AtC_ were estimated by matching the model-predicted rate of oxygen consumption, MVO_2_, to the reported experimentally measured estimates of MVO_2_. Circles, squares, and triangles represent data from pacing protocol (Table 2 of [5]), epinephrine protocol (Table 3 of [5]), and phenylephrine protocol (Table 4 of [5]), respectively. The solid line represents the best fit to the data, *G* = 0.3326 + (2.315)*J*_AtC_, where *J*_AtC_ is expressed in units of mmol s^−1^ (l cell)^−1^ and *G* is computed in units of ml min^−1^ (g of tissue)^−1^. (B) The predicted ratio between mitochondrial ATP production and rate of oxygen atom consumption (P/O ratio) is plotted for *J*_AtC_ values from 0 to 1.7 mmol s^−1^ (l cell)^ −1^. At *J*_AtC_ = 0, the predicted MVO_2_ is 1.68 μmol min^−1^ (g of tissue)^−1^, corresponding to the oxygen consumption rate necessary to offset rate of proton leak across the inner mitochondrial membrane.

**Table 2:**
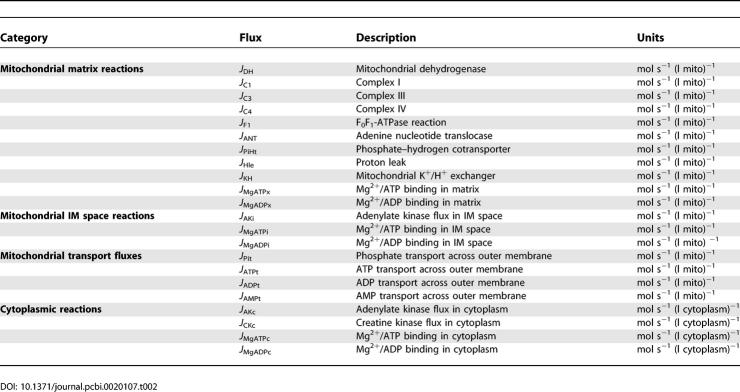
Model Fluxes

**Table 3:**
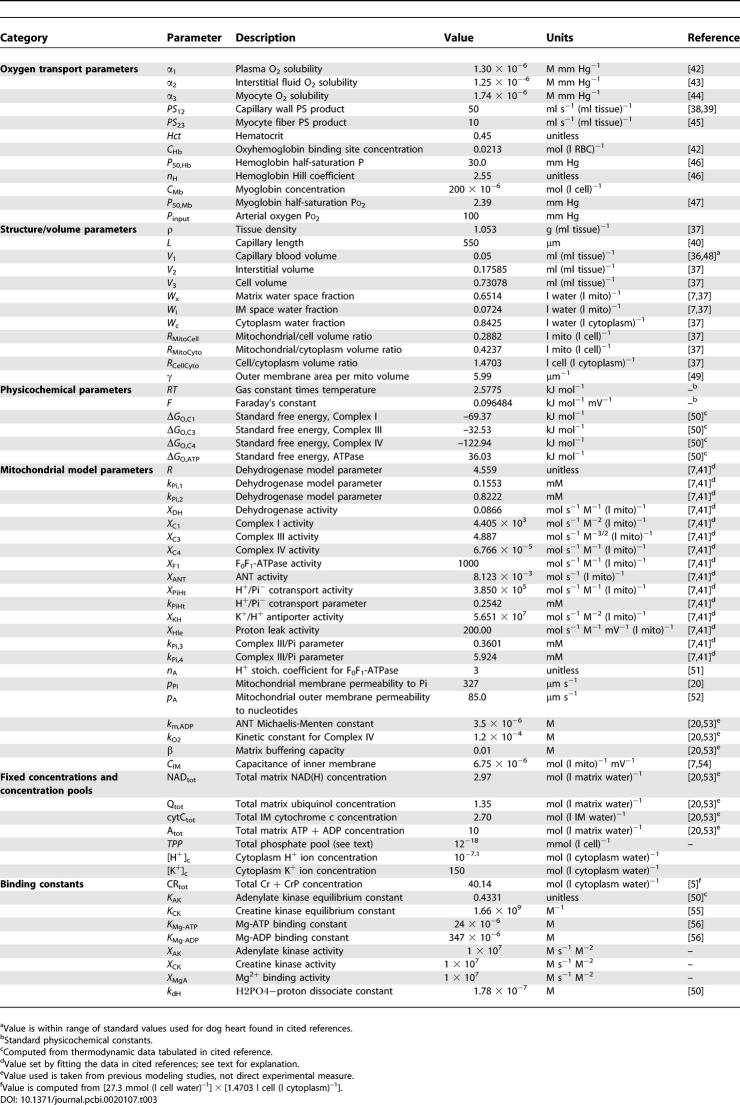
Parameter Values

Figure 2B illustrates the predicted relationship between mitochondrial ATP production and oxygen consumption over the range a range of *J*_AtC_ values from 0 to 1.7 mmol s^−1^ (l cell)^ −1^. The P/O ratio is the ratio of the rate of ATP synthesis by the F_0_F_1_-ATPase to the rate of consumption of oxygen atoms. At *J*_AtC_ = 0, the P/O ratio is 0 because no ATP is synthesized while a finite oxygen consumption flux exists to offset the rate of proton leak across the inner mitochondrial membrane. At high values of ATP consumption (and oxygen consumption), the P/O ratio approaches the theoretical maximum of 2.50 for the oxidative phosphorylation model of Beard [7].

### Phosphate Metabolites as a Function of Workload

Using the developed linear relationship between workload and coronary flow, steady-state model predictions were calculated for a range of *J*_AtC_ values from 0 to 1.7 mmol s^−1^ (l cell)^ −1^. Over this range, the predicted MVO_2_ varies from 1.68 μmol min^−1^ (g of tissue)^−1^ to 15.7 μmol min^−1^ (g of tissue)^−1^. Predictions of ADP and Pi concentrations and CrP/ATP ratio as functions of MVO_2_ are plotted in Figure 3. In Figure 3, concentrations of metabolites are computed as the average value in the distributed model for a given steady-state condition.

**Figure 3:**
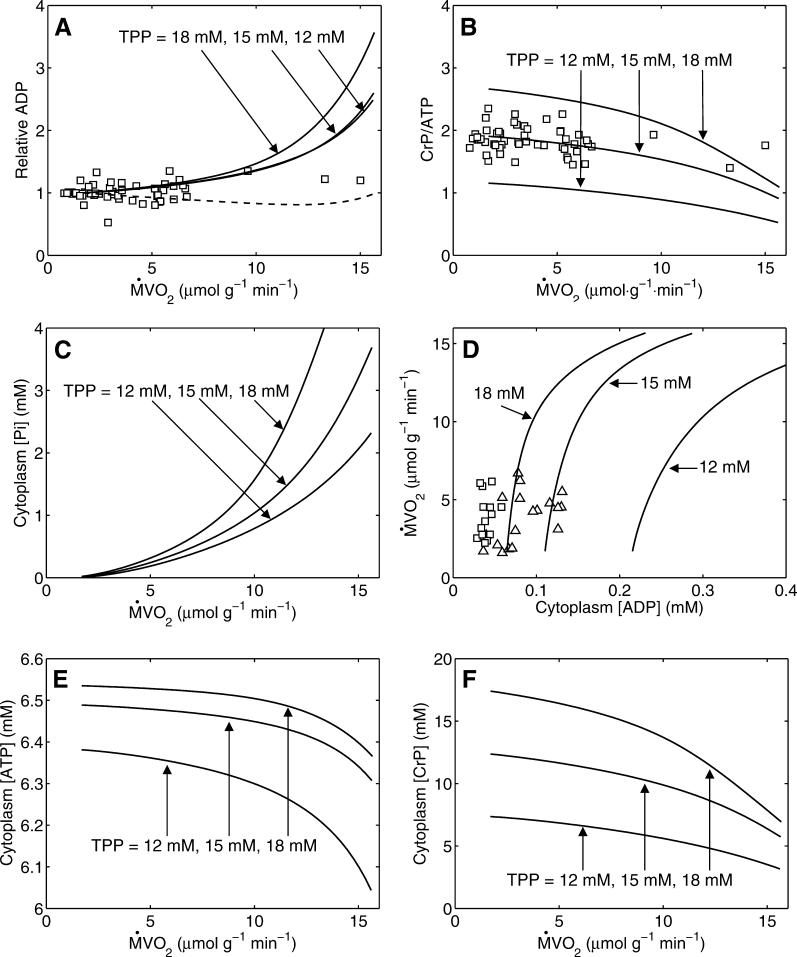
Comparison of the Model Simulations to Experiment Data on Energetic Phosphate Compounds in Cardiac Tissue at Varying Cardiac Work Rate (A) Normalized ADP is plotted as a function of MVO_2_. Solid lines correspond to model simulations of cytoplasmic ADP at different values of total phosphate pool, *TPP* = 12, 15, and 18 mM. Dashed line represents total relative ADP mass (cytoplamic plus mitochondrial) predicted for *TPP* = 15 mM. (B) Ratio cytoplasmic CrP/ATP is plotted as a function of MVO_2_. Solid lines correspond to model simulations at different values of total phosphate pool, *TPP* = 12, 15, and 18 mM. (C) Cytoplasm Pi concentration is plotted as a function of MVO_2_. (D) Predicted MVO_2_ is plotted as a function of predicted ADP concentration in cytoplasm. Solid lines correspond to model simulations at different values of total phosphate pool, *TPP* = 12, 15, and 18 mM, indicated in figure. Experimental data in (A) and (B) are obtained from Katz et al. [5]; data in (D) is obtained from Portman et al. [9]. In all panels, model curves are obtained by varying *J*_AtC_ from 0 to 1.7 mmol s^−1^ (l cell)^ −1^ and setting flow according to *G* = 0.3326 + (2.315)*J*_AtC_, where *J*_AtC_ is expressed in units of mmol s^−1^ (l cell)^−1^ and *G* is computed in units of ml min^−1^ (g of tissue)^−1^. (E) Predicted cytoplasmic ATP concentration is plotted as a function of predicted MVO_2_. (F) Predicted cytoplasmic CrP concentration is plotted as a function of predicted ADP concentration in cytoplasm.

The black curves in Figure 3A represent the relative cytoplasmic ADP level (normalized to the value at *J*_AtC_ = 0) as a function of MVO_2_ for three different values of the total phosphate pool parameter (see Equation 26 in Materials and Methods). Also plotted are data from Figures 2A, 4A, and 6A of Katz et al. [5], which represent experimental observations for the three different stimulus protocols described above. It is apparent that within the range of observed variability, the model predictions match the experimental data for values of MVO_2_, less than approximately 12 μmol min^−1^ (g of tissue)^−1^. At the highest simulated level of *TPP* (18 mM), the model predictions diverge from the observed data at MVO_2_ values slightly lower than is predicted at *TPP* equal to 12 mM and 15 mM. However, regardless of the value of *TPP,* the model predicts that the relative ADP concentration is 2-fold to 3-fold higher at MVO_2_ = 15 μmol min^−1^ (g of tissue)^−1^ than at the lower work rates. Although the amount of available data for extremely high workloads near MVO_2_ = 15 μmol min^−1^ (g of tissue)^−1^ is sparse, the observations indicate that even at these extreme work rates, the cellular ADP level does not significantly increase from the minimum value.

**Figure 4:**
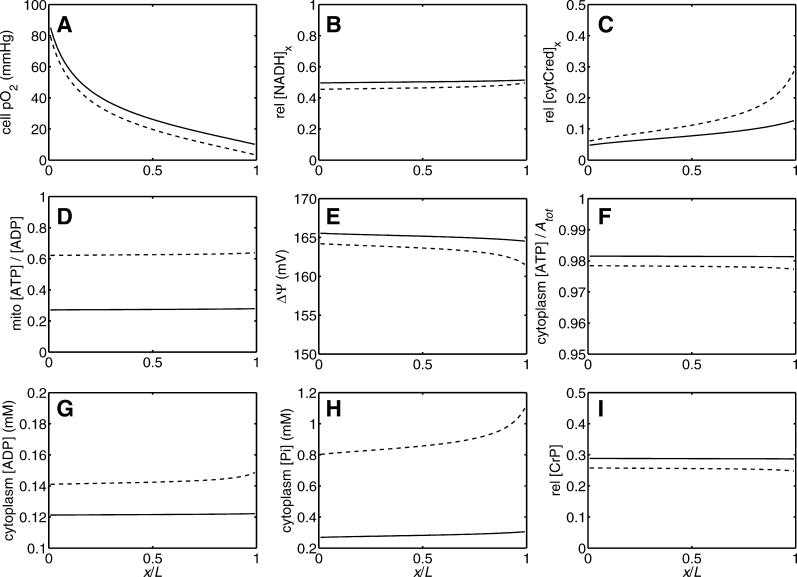
Spatial Profiles of Model Variables at Moderate and High Work Rates Model variables are plotted as a function of *x*/*L,* the normalized distance along the capillary. Arterial blood flows into the capillary at *x* = 0. For all panels, solid line corresponds to moderate work rate; dashed line corresponds to high work rate. Moderate work rate is defined by setting *J*_AtC_ = 0.45 mmol s^−1^ (l cell)^ −1^ and *G* = 0.75 ml min^−1^ (g of tissue)^−1^; high work rate is defined by setting *J*_AtC_ = 0.90 mmol s^−1^ (l cell)^ −1^ and *G* = 1.25 ml min^−1^ (g of tissue)^−1^. (A) Cellular pO_2_ is plotted versus *x*/*L*. (B) Mitochondrial NADH/NAD_tot_ is plotted versus *x*/*L*. (C) Mitochondrial cytoC(red)^2+^/cytC_tot_ is plotted versus *x*/*L*. (D) Mitochondrial ATP/ADP is plotted versus *x*/*L*. (E) Mitochondrial membrane potential is plotted versus *x*/*L*. (F) Cytoplasm ATP/A_tot_ is plotted versus *x*/*L*. (G) Cytoplasm ADP concentration is plotted versus *x*/*L*. (H) Cytoplasm Pi concentration is plotted versus *x*/*L*. (I) Cytoplasm CrP/CR_tot_ is plotted versus *x*/*L*.

Figure 3B plots the predicted CrP/ATP ratio in the cytoplasm as a function of MVO_2_, as well as the data from Figures 2C, 4C, and 6C of Katz et al. [5]. Model predictions at *TPP* = 15 mM are most consistent with the experimental data. At this value for the total phosphate pool, the model predicts that the ratio CrP/ATP drops from 2 at low work rate to approximately 1 at the highest work rate. Over the range of MVO_2_ from 2 to 6 μmol min^−1^ (g of tissue)^−1^, the predicted CrP/ATP ranges from 1.89 to 1.76 at *TPP* = 15 mM.

Model-predicted concentrations of cytoplasmic Pi as a function of work rate are plotted in Figure 3C. The model predicts that at *TPP* = 15 mM, [Pi]_c_ increases from 0.02 mM at 0 workload to 0.36 mM at MVO_2_ = 6 μmol min^−1^ (g of tissue)^−1^ to 3.1 mM at MVO_2_ = 15 μmol min^−1^ (g of tissue)^−1^. Over the range for which the model best fits the observed data on ADP and CrP/ATP [MVO_2_ = 1.68 to 12 μmol min^−1^ (g of tissue)^−1^], the model predicts that Pi concentration increases from 0.02 mM to 1.6 mM. While it is difficult to absolutely quantify Pi concentration in vivo in the heart from ^31^P-NMR spectroscopy, it is possible to estimate changes in the amount of Pi relative to other phosphate metabolites. Zhang et al. [14] report ΔP/CrP in the canine heart—the change in Pi relative to phosphocreatine—from baseline to maximally stimulated contraction to be 0.21. This value of ΔP/CrP corresponds to a change in Pi of approximately 2 mM, which is consistent with our model predictions.

In Figure 3D, the predicted MVO_2_ is plotted as a function of absolute cytoplasmic ADP concentration. For comparison, data obtained from in vivo hearts of newborn (open squares) and adult (shaded triangles) from Portman et al. [9] are plotted in Figure 3D. Cytoplasmic ADP concentration is predicted to be approximately 0.1 to 0.15 mM in low and moderate workload conditions at the preferred value of *TPP* = 15 mM.

Figure 3E illustrates that cytoplasmic ATP concentration remains constant (with less than 10% variation) over the simulated range of work rate for all values of *TPP*. Thus the predicted variation in CrP/ATP ratio is due to changes in the CrP concentration, as illustrated in Figure 3F, which plots cytoplasm CrP as a function of MVO_2_.

Based on the above calculations, the parameter *TPP* is set to 15 mM for the remaining simulations.

### Cytoplasmic versus Mitochondrial Metabolite Pools

As described above, the solid curves in Figure 3 represent the predicted levels of ADP, CrP/ATP, and Pi in the cytoplasm. Comparing these values to the data of Katz et al. [5] is consistent with assumptions made in that paper that an experimental estimate for cytoplasmic ADP concentration can be obtained from the equation [ADP] = [ATP][Cr]/*K*_CK_[CrP][H^+^]. The application of this equation to experimental data on [ATP] and [CrP]/[Cr] is based on two assumptions: 1) that the measured levels of both ATP and CrP represent concentrations in the cytoplasm and 2) that the creatine kinase (CK) reaction is maintained in equilibrium. This standard assumption is invoked, for example, in the studies of Balaban and coworkers [2,5,9]. However, Garlick and coworkers [15–17] find that both the cytoplasmic and mitochondrial ATP pools are visible in ^31^P NMR spectroscopy. If this were the case, then it would not be appropriate to assume that the total adenine nucleotide pool is in chemical equilibrium with the Cr and CrP, and consequently the reported relative ADP concentrations would not represent unbiased estimates of cytoplasmic ADP. The current model predicts that the total (cytoplasmic plus mitochondrial) ADP pool (plotted as dashed line in Figure 3A) is effectively constant over the full range of work rate simulated.

### Predicted State Variables at Different Workloads

While the previous section illustrates the spatially averaged cardiac phosphate metabolite levels at different steady-state work rates, in this section predicted spatial profiles of model variables are explored at two different work rates. Specifically, simulations were performed at *J*_AtC_ = 0.45 mmol s^−1^ (l cell)^ −1^ and *G* = 0.75 ml min^−1^ (g of tissue)^−1^ (defined as *moderate* work rate) and at *J*_AtC_ = 0.90 mmol s^−1^ (l cell)^ −1^ and *G* = 1.25 ml min^−1^ (g of tissue)^−1^ (defined as *high* work rate). At the moderate work rate, oxygen consumption and extraction were computed to be 5.3 μmol min^−1^ (g of tissue)^−1^ and 76%; at the high work rate, oxygen consumption and extraction were computed to be 9.1 μmol min^−1^ (g of tissue)^−1^ and 78%. The predicted values of venous Po_2_ are 18.5 mm Hg and 17.9 mm Hg for the moderate and high work rates, respectively.

Predicted spatial profiles of nine state variables are plotted in Figure 4, with solid lines and dashed lines representing data from the moderate and high work rates, respectively. From Figure 4A, it is apparent that at the high work rate, cellular Po_2_ approaches 0 near the venous end of the capillary. At the moderate work rate, the minimum cellular Po_2_ is 10.6 mm Hg; the minimum cellular Po_2_ at the high work rate is 2.9 mm Hg. Although the cellular Po_2_ varies along the length of the capillary, it is apparent that at a given workload, most of the state variables remain relatively constant throughout the cell region. Mitochondrial NADH and cytoplasmic ADP and Pi are predicted to be slightly elevated in the region of lowest oxygenation at the high work rate; ΔΨ is 2 mV to 3 mV lower in the region of low oxygen at the high work rate than it is for the majority of the myocyte space. The cytoplasmic ATP concentration (Figure 4F) is approximately 98% of the total adenine nucleotide pool at moderate and high work rates. The cellular Pi concentration is predicted to increase from approximately 0.3 mM at moderate work to approximately 1 mM at high work.

### Phosphate Metabolites during Graded Hypoperfusion

To explore the behavior of the integrated model during ischemia, when the tissue may become hypoxic to an extent greater than that observed in the simulations described above, it is not possible to treat the ATP consumption flux as a constant that does not depend on the available cytoplasmic ATP. To ensure that ATP concentration is not allowed to become negative, it is necessary to model the ATP consumption flux using an expression that approaches 0 as ATP concentration approaches 0. In this section, in which the high-energy phosphate metabolic response of cardiac tissue to graded hypoperfusion is simulated, the ATP consumption flux is modeled using the expression

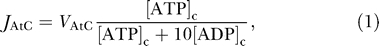
where the flux approaches the constant *V*_AtC_ under normal conditions when ATP concentrations are more than 50 times higher than cytoplasm ADP concentrations.

To simulate the model response to graded hypoperfusion, *V*_AtC_ is set to 0.48 mmol s^−1^ (l cell)^ −1^ and flow is varied from 0.025 to 1.25 ml min^−1^ (g of tissue)^−1^, corresponding to the range of coronary blood flows imposed in the study by Zhang et al. [10]. The value *V*_AtC_ = 0.48 mmol s^−1^ (l cell)^ −1^ was chosen to achieve reasonable agreement between the experimental data and model predictions. Over the range of myocardial flow, Zhang et al. [10] measured ATP, CrP, Pi, and oxymyoglobin saturation (*S*_Mb_), in dog myocardium in vivo. These data are compared to steady-state model predictions in Figure 5. The raw data from Zhang et al. [10] were normalized so that the measured ATP concentration at normal flow was equal to 6.5 mM, the model-predicted ATP concentration for normoxic nonischemic conditions. The same scaling that was applied to the raw data for ATP was applied to the raw CrP and Pi data to obtain estimates of the molar concentrations of these species.

**Figure 5:**
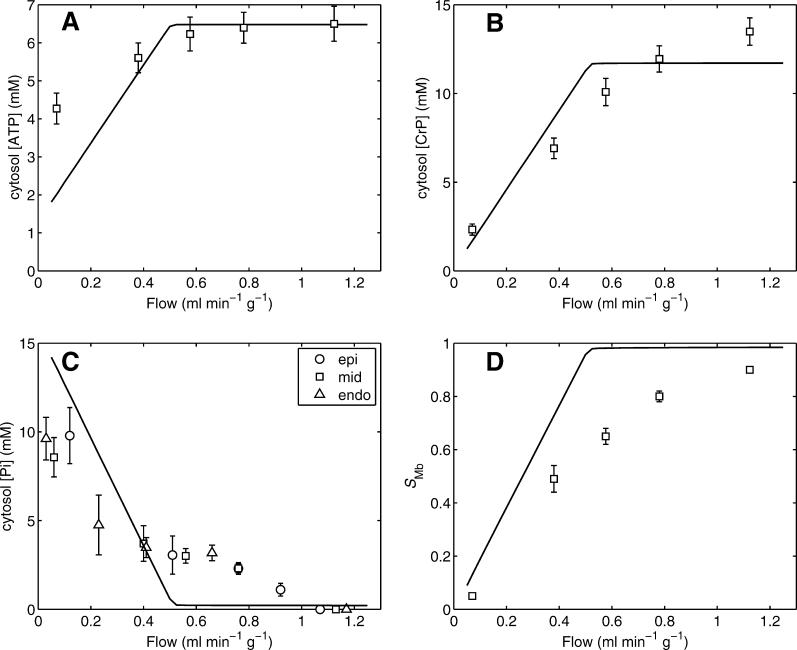
Model Simulations of Cardiac Energetic Phosphate Metabolite Levels and Myoglobin Saturation during Coronary Hypoperfusion (A) Model-predicted cytoplasmic ATP concentration as a function of myocardial blood flow. (B) Model-predicted cytoplasmic CrP concentration as a function of myocardial blood flow. (C) Model-predicted cytoplasmic Pi concentration as a function of myocardial blood flow. Experimental data are divided into epicardium (epi), midwall (mid), and endocardium (endo). (D) Model-predicted mean myoglobin saturation as a function of myocardial blood flow. For all panels, ATP consumption flux is simulated using Equation 1, and *V*_AtC_ is set to 0.48 mmol s^−1^ (l cell)^ −1^. Experimental data are for canine and left ventricular myocardium and obtained from Zhang et al. [10].

As expected, ATP, CrP, and *S*_Mb_ levels drop and cytoplasm Pi increases as flow is reduced. In general, the order of magnitude and the qualitative trends in the model predictions match the experimental observations. However, the experimental observations show a smoother variation with flow in these four variables than is predicted by the model. The observed differences between model predictions and observed data of Figure 5 are expected to be in part accounted for by the fact that in the current model myocardial heterogeneities in flow and capillary length are not considered. This issue is discussed in greater detail in the Discussion section.

## Discussion

### Control of Phosphate Metabolites

The major finding of this study is that the integrated model is able to reproduce experimental observations on ATP, ADP, CrP, and Pi over a range of workload and during coronary hypoperfusion. The integrated model explains metabolite levels observed at low to moderate workloads and the changes in metabolite levels and tissue oxygenation observed during graded hypoperfusion. The level of agreement between model predictions and experimental measurements is significant because the integrated model introduces only a single adjustable parameter. During a 10-fold increase in workload (rates of ATP and oxygen consumption), the cytoplasmic ATP, ADP, and CrP concentrations and mitochondrial NADH concentrations are predicted to change little. The cytoplasmic Pi concentration is predicted to increase from approximately 0.15 mM at an oxygen consumption of 4 μmol min^−1^ (g of tissue)^−1^ to approximately 1.0 mM at an oxygen consumption of 10 μmol min^−1^ (g of tissue)^−1^. Over this range the model predictions closely match experimental measurements and indicate that cytoplasmic Pi level is a key regulator of the rate of mitochondrial respiration. At work rates higher than approximately 12 μmol min^−1^ (g of tissue)^−1^, the model predictions of cytoplasmic ADP and CrP/ATP ratio are slightly elevated compared to low work states. Total cytoplasmic plus mitochondrial ADP is effectively constant over the entire observed range of work rate. In addition, the validated model provides quantitative predictions of changes in phosphate metabolites during cardiac ischemia. Comparison of model predictions to the data of Zhang et al. [10] provides independent validation of the model. Although these modeling results depend on the chosen value of the maximal rate of ATP consumption (*V*_AtC_), the model predictions in the extreme limits of flow do not depend on the choice of *V*_AtC_. It is significant that the predicted Pi concentration of approximately 14 mM in the zero-flow limit and the predicted CrP concentration of approximately 12 mM at the high-flow limit are close to the observations of Zhang et al. [10].

Previous modeling studies of Korzeniewski et al. [18] have attributed the myocardium's ability to maintain homeostatic concentrations of ATP and CrP over a range of work rates to a “parallel activation” mechanism in which all respiratory complexes and the rate of substrate dehydrogenation are increased in synchrony with the rate of ATP consumption. However, no independent verification of the parallel activation hypothesis exists. In the current analysis, the biophysical model of cardiac oxidative phosphorylation and mitochondrial substrate transport was developed from an entirely independent set of data on isolated mitochondria [7]. The observed stability of energy metabolites emerges as a property of a properly constructed model of cardiac oxygen transport and mitochondrial metabolism. Thus the current analysis casts some doubt on the parallel activation hypothesis.

As concluded previously by Saks et al. [19,20], the current analysis predicts that Pi is an important feedback signal regulating respiration in cardiomyocytes. However, important differences exist between the current model and earlier models presented by the Saks group [20,21]. Specifically, the earlier models have applied an arbitrary scaling to previously developed models of mitochondrial oxidative phosphorylation, to match the observed dynamic range of oxygen consumption in isolated rat heart. The current model scales the model of isolated mitochondria based on independent estimates of cardiac mitochondrial volume density, eliminating an arbitrary fitting parameter necessary in the earlier models. Studies from the Saks laboratory have also suggested that coupling between mitochondrial creatine kinase and the adenine nucleotide translocase system on the mitochondrial inner membrane plays a role in maintaining metabolic homeostasis in the heart [20–23]. It will be valuable to incorporate the Vendelin–Saks model of the mitochondrial creatine kinase shuttle into the current model to explore its possible role in regulating respiration and metabolite concentrations.

The findings of the current study are consistent with the observations of Jeneson et al. [24,25] that, in skeletal muscle, ADP concentrations vary less than approximately 5-fold between minimum and maximal work rate. The current model predicts that ADP is 1.3 times higher at MVO_2_ of 10 μmol min^−1^ (g of tissue)^−1^ than at MVO_2_ of 1.7 μmol min^−1^ (g of tissue)^−1^. At the maximal MVO_2_ the predicted ADP concentration is 2.5 times greater than that at the minimum. The predicted cardiac Pi concentration is predicted to be too low to be detectable in NMR at low work rates [0.15 mM at MVO_2_ of 4 μmol min^−1^ (g of tissue)^−1^] and to increase to the millimolar range at high work rates [1.0 mM at MVO_2_ of 10 μmol min^−1^ (g of tissue)^−1^].

### Mitochondrial P/O Ratio

The oxidative phosphorylation model predicts that the cardiac mitochondrial P/O ratio exceeds the value of 2 for MVO_2_ greater than 7.8 μmol min^−1^ (g of tissue)^−1^ or approximately 200 μl O_2_ min^−1^ (g of tissue)^−1^. For lower values of oxygen consumption, the P/O ratio is less than 2, indicating that oxidative ATP synthesis is significantly less efficient at moderate work rates than at maximal work rates in the heart. This behavior is due to the fact that the predicted oxygen flux necessary to compensate for proton leak is nearly constant. Thus the relative contribution of proton leak to oxygen consumption increases with decreasing MVO_2_.

Since the oxygen flux associated with the proton leak is approximately one tenth of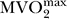 , the maximal rate of oxygen consumption, the P/O ratio as a function of MVO_2_ is approximately expressed as P/O = 2.5 (MVO_2_ − 0.1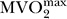 )/ MVO_2_, where 2.5 is the theoretical maximum obtained by the model of oxidative phosphorylation in the absence of a leak current.

### Future Considerations

It is likely that additional regulatory mechanisms that are not currently incorporated into the model may become important at high work rates. One putatively important signal is calcium. Cortassa et al. [26] and Jafri et al. [27–29] developed models of mitochondrial calcium handling and calcium-dependent activation of the TCA cycle. At high work rates, increased mitochondrial calcium may provide a feed-forward signal to stimulate increased dehydrogenase activity. The current model, which uses a phenomenological function to drive the reduction of NAD^+^, does not account for the possible role of calcium signaling in controlling cardiac respiration. Future work will integrate the detailed kinetics of the TCA cycle and calcium activation of the Jafri and Cortassa models into the current model to explore the impact of calcium signaling on cardiac metabolic regulation.

Previous analysis of oxygen transport in isolated perfused hearts has revealed that heterogeneities in microvascular geometry and flow are important in realistically simulating oxygen transport to cardiac tissue [30,31]. Yet the current model uses a single-capillary three-region model to simulate oxygen transport and metabolism. It is likely that the lack of heterogeneity in the model is responsible in part for the deviations between model predictions and experimental measurements of ATP, Pi, CrP, and *S*_Mb_ during hypoperfusion. The predicted sharp transition from constant metabolite concentrations at higher flows to linearly decreasing concentrations at lower flows is a result of the model being composed of a single capillary. When flow is high enough to maintain normoxia, the energy metabolite concentrations are predicted to be stable. At lower flows, the myoglobin saturation is directly proportional to the fraction of the cell that is normoxic, which increases linearly with flow at a constant work rate. In the future, the current detailed model of cellular energetics will be ported to simulations in heterogeneous three-dimensional geometries, such as that described in [30]. The single-capillary model is used in the current work because the computational costs of the integrated model are substantial. Conducting such simulations based on a three-dimensional model will require significant computational times, substantial optimization of the simulation codes, and/or increases in available computing resources.

The current model analysis of the data on flow and oxygen consumption reveals an approximately linear relationship between whole-heart cardiac perfusion and the rate of ATP consumption (illustrated in Figure 2A). Yet it has been observed that local myocardial blood flow can vary regionally by up to 10-fold in vivo [32–35]. Since oxygen [35] and fatty acid [36] uptake are tightly proportional to regional flow in the myocardium, it is proposed that ATP hydrolysis varies regionally as well. Future studies that link coronary blood flow, oxygen and substrate transport, cellular and whole-heart mechanics, and energy metabolism are expected to shed light on the physiological basis for regional heterogeneities in substrate delivery in the heart.

### Summary

In sum, the integrated model of cardiac oxygen transport and mitochondrial oxidative phosphorylation is able to explain a set of data that has persisted unexplained in the literature for two decades. The model analysis predicts that Pi is a key regulator of oxidative phosphorylation in the heart, allowing ATP and CrP levels to remain relatively constant over a large range of cardiac work rate. The integrated model provides the basic framework for simulating the metabolic response of the myocardium to ischemia and hypoxia.

## Materials and Methods

### Governing equations for oxygen transport.

Oxygen transport in the capillary is governed by advection of the blood, oxygen–hemoglogin binding and unbinding, and passive permeation between the blood and the interstitial space. The governing equation for oxygen in the capillary is

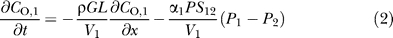
where *C*_O,1_(*x*,*t*) is the total oxygen concentration in the blood (free plus hemoglobin-bound oxygen), *G* is blood flow to the tissue in volume per unit time per mass of tissue, ρ is the tissue density in mass per unit volume, *L* is the length of the capillary, *V*_1_ is the volume of the capillary region, *α*_1_ is the oxygen solubility coefficient in blood, *PS*_12_ is the permeability of the surface area of capillary wall, and *P*_1_ and *P*_2_ are the oxygen partial pressures in blood and interstitial fluid, respectively. The capillary oxygen concentration *C*_O,1_ is a function of the distance along the capillary, *x*, and time, *t*.

The total oxygen concentration of the capillary region is related to the partial pressure by

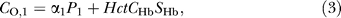
where *Hct* is the hematocrit, *C*_Hb_ is the concentration of oxygen binding sites in red blood cells, and *S*_Hb_ is the hemoglobin saturation in red blood cells. The hemoglobin saturation curve is assumed to be governed by a Hill equation,

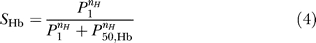
where *P*_50,Hb_ is half the partial pressure of O_2_ saturation in hemoglobin and *n_H_* is the Hill exponent.

Oxygen transport in the interstitial region is governed by passive permeation of oxygen between the blood and the interstitial fluid and between the interstitial fluid and the myocyte

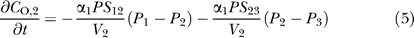
where *C*_O,2_(*x*,*t*) is the oxygen concentration in interstitial region, *V*_2_ is the volume of the interstitial region, *PS*_23_ is the permeability surface-area product for passive permeation between interstitium region and the parenchymal cell region, and *P*_3_ is the partial pressure of the oxygen in the cell. Oxygen concentration and partial pressure in the interstitial fluid are related by *C*_O,2_ = *α*_2_ *P2*, where *α*_2_ is the oxygen solubility coefficient in the interstitial region.

In the cellular region, oxygen transport is governed by passive permeation and consumption of oxygen in mitochondria

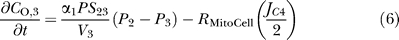
where *C*_O,3_ is the oxygen concentration in the cellular region, *V*_3_ is the volume of the cellular region, *R*_MitoCell_ is the ratio of mitochondrial volume to total cell volume, and *J*_C4_ is the flux through Complex IV in the respiratory chain in units of mass per time per unit mitochondrial volume. The factor of ½ in the second term on the RHS of Equation 6 accounts for the fact that *J*_C4_ in the mitochondrial model (described below) represents the flux of electron pairs through the respiratory chain: one O_2_ molecule is consumed for each two pairs of electrons transferred through the system. Equation 6 assumes that mitochondria are uniformly and homogeneously distributed in the cellular space.

Total oxygen in the cell is the sum of free oxygen plus myoglobin-bound oxygen

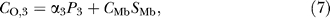
Where *C*_Mb_ is the myoglobin concentration in the cell and *α*_3_ is the oxygen solubility in the myocyte. The equilibrium myoglobin saturation *S*_Mb_ is expressed

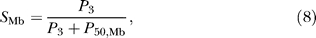
where *P*_50,Mb_ is the half-saturation partial pressure for oxygen–myoglobin binding.

### Governing equations for cellular energetics.

The differential equations for species other than oxygen are

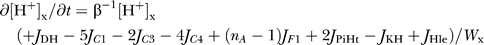


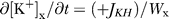


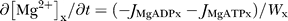


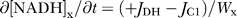


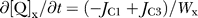


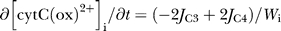


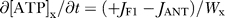


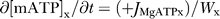


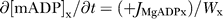


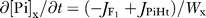


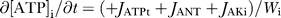


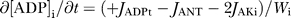


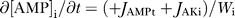


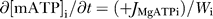


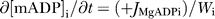


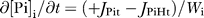


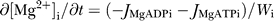


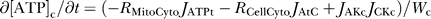


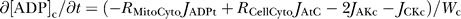


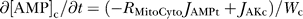


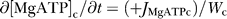


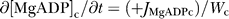


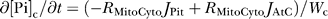


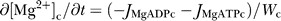


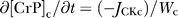


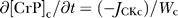


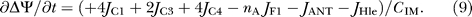



In the above set of equations, the subscripts x, i, and c denote mitochondrial matrix, intermembrane (IM) space, and cytoplasm, respectively. All of the variables in this set of equations are defined in Table 1.

In addition to the state variables treated in Equations 1, 4, 5, and 8, the concentrations of several species are computed

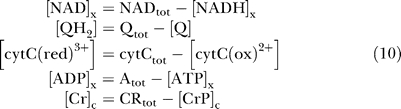
where NAD_tot_, Q_tot_, cytC_tot_, and A_tot_ are the total concentrations of NAD(H), ubiquinol, cytochrome c, and adenine nucleotide in the matrix, respectively, and CR_tot_ is the total creatine plus creatine phosphate concentration in the cytoplasm.

Parameters that appear in the above equations are described in detail below. The fluxes that appear on the right-hand side of the governing equations are tabulated in Table 2. For mitochondrial species, the governing equations follow from [7]. For cytoplasmic species, the reactions modeled are ATP consumption, creatine kinase reaction, adenylate kinase reaction, and transport between the cytoplasm and the mitochondrial IM space.

### Mathematical expressions for mitochondrial fluxes.

Flux expressions for the mitochondrial model (based on a previously developed model [7]) are listed below. Definitions of the variables and parameters that appear in the following expressions are listed in Tables 2 and 3.

Dehydrogenase flux:

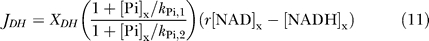



Complex I flux:

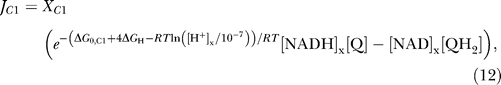
where Δ*G*_H_ = *F*ΔΨ + *RT*1n([H^+^]_c_/H^+^]_x_).

Complex III flux:

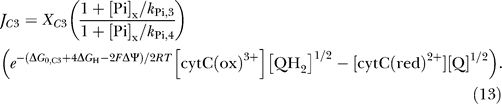



Complex IV flux:

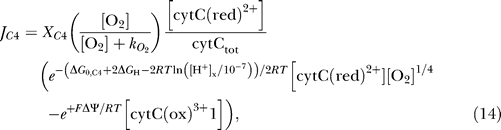
where [O_2_] = *α*_3_ *P*_3_ is the free oxygen concentration in the cell.

F_0_F_1_-ATPase flux:

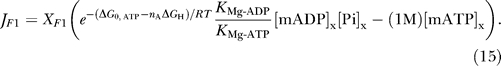



Magnesium binding fluxes:

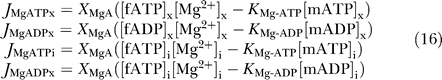
where [fATP]_x_, [fADP]_x_, [fATP]_i_, and [fADP]_i_ denote magnesium unbound ATP in the matrix, ADP in the matrix, ATP in the IM space, and ADP in the IM space, respectively.

Substrate transport fluxes:

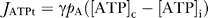


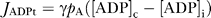


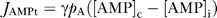


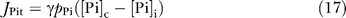



Adenine nucleotide translocase (ANT) flux:

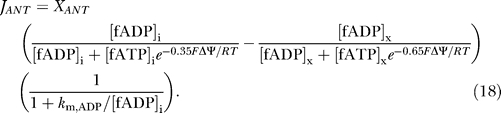



The phosphate–hydrogen cotransporter flux:

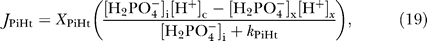
where [H_2_ 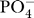 ]_i_ = [H^+^]_i_[Pi]_i_/([H^+^]_i_ + *k*_dH_) and [H_2_ 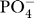 ]_x_ = [H^+^]_x_[Pi]_x_/([H^+^]_x_ + *k*_dH_).

Mitochondrial adenylate kinase flux:

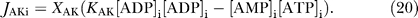



Proton leak flux:

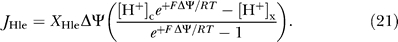



Potassium–hydrogen ion exchange:

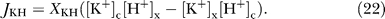
The above expressions for the mitochondrial model fluxes are identical to those presented in [7], with the exception of the Complex I and III fluxes, which have been modified to ensure that the model remains numerically stable when [O_2_] and [Q] reach 0.

### Mathematical expressions for cytoplasmic reaction fluxes.

Four biochemical processes are modeled in the cytoplasm—the adenylate kinase reaction, the creatine kinase reaction, ATP hydrolysis, and binding of magnesium ions to ADP and ATP.

The binding of magnesium to ATP and ADP in the cytoplasm takes the same form as the binding fluxes in the mitochondria:

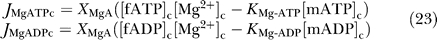
where [fATP]_c_ and [fADP]_c_ denote magnesium unbound ATP and ADP in the cytoplasm. Similarly, the cytoplasmic adenylate kinase is analogous to the mitochondrial reaction.

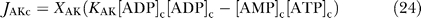



In Equation 24, *K*_AK_ is the equilibrium constant for the reaction 2 ADP ATP + AMP, and *X*_AK_ is the enzyme activity, which is set to a large enough value so that the reaction is effectively maintained in equilibrium.

The creatine kinase flux is modeled using the expression:

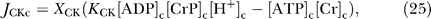
where the activity *X*_CK_ is set to a large enough value so that the equilibrium *K*_CK_ = ([ATP]_c_[Cr]_c_/[ADP]_c_[CrP]_c_[H^+^]_c_)_eq_ is maintained during simulations.

The flux *J*_AtC_ is defined as the flux through the reaction ATP → ADP + Pi. Mathematical models for the ATP consumption flux are considered in the Results section.

### Parameter values.

With the exception of one adjustable parameter, all parameters in the model are fixed at values justified by previous studies. The adjustable parameter is the total pool of exchangeable phosphate in the cell, which is a constant denoted by *TPP*. The total exchangeable phosphate pool is computed as

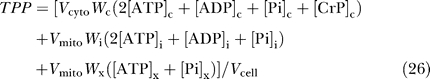
where *V*_cyto_ = 0.472 ml (g of tissue)^−1^, *V*_mito_ = 0.200 ml (g of tissue)^−1^, and *V*_cell_ = 0.694 ml (g of tissue)^−1^ are the volume densities of cytoplasm, mitochondria, and total cell space in cardiac tissue [37]. By comparing simulation predictions with experimental data (see Results section), a value of *TPP* = 15 mM is chosen as the value most consistent with the experimental observations.

The parameter values listed in Table 3 are organized into oxygen transport parameters, structure/volume parameters, physicochemical parameters, mitochondrial model parameters, fixed concentration pools, and binding constants.

The oxygen transport parameters, including solubilities, permeabilities, and oxyhemoglobin and oxymyoglobin binding parameters, are obtained from the literature, as indicated in the table. The effective permeability–surface area product for the capillary wall is obtained by converting the mass transfer coefficient reported in [38] and used in [39] to the corresponding *PS* product. Similarly, the structure/volume parameters are available from experimental estimations published in the literature. The effective capillary length is set to the mean capillary path length for left ventricular tissue reported by Kassab and Fung [40]. The majority of the volume density and water space measurements are obtained from the study of Vinnakota and Bassingthwaighte [37]. Values of the mitochondrial model parameters are estimated and reported in [7].

Oxidative phosphorylation model parameters have been adjusted to account for modifications from [7], as indicated in the table. Since the F_0_F_1_-ATPase reaction is maintained near chemical equilibrium in the model parameterization that was published in [7], here the F_0_F_1_-ATPase activity *X*_F1_ is set to the arbitrarily high value of 1,000 mol s^−1^ M^−1^ (l mito)^−1^. In addition, the updates to the functional forms for the Complex I and III fluxes require estimation of values for the activities of *X*_C1_ and *X*_C3_. To account for these changes, the full set of mitochondrial model parameters has been refined by repeating the fits to data from isolated mitochondrial function of Beard [7]. The agreement between the data of Bose et al. [41] and the updated model is equivalent to that reported for the previous version of the model [7].

All values for concentrations of pooled metabolites are set according to values reported in previous studies, with the exception of the total pool of exchangeable phosphate (*TPP*), which is estimated in the Results section. Binding constants are obtained from the literature; enzyme activities for reactions maintained near equilibrium are set to arbitrarily high values.

### Computational methods.

The model was coded using MATLAB; multiple steady-state solutions were obtained using the Matlab Distributed Computing Toolbox on 25-node cluster (50-CPU AMD Opteron-based server HP DL145 G2 with dual 2.6-GHz processors). Obtaining a single steady-state solution requires approximately 30 min of computing time on a single processor. A total of 150 steady-state solutions are computed to construct Figure 4, requiring less than 2 h to complete using the cluster.

## Abbreviations

ADP: adenosine diphosphate
AMP: adenosine monophosphate
ATP: adenosine triphosphate
CK: creatine kinase
CrP: creatine phosphate
IM: intermembrane
Pi: inorganic phosphate
S_Mb_: oxymyoglobin saturation
V_AtC_: rate of ATP consumption
